# Is Congenital Talipes Equinovarus a Risk Factor for Developmental Dysplasia of the Hip?

**DOI:** 10.3390/jcm15218469

**Published:** 2026-10-30

**Authors:** Durmus Ekin Dincer, Nusret Köse

**Affiliations:** 1 Department of Orthopedics and Traumatology, Gaziantep City Hospital, Gaziantep 27470, Turkey; 2 Department of Orthopedics and Traumatology, Faculty of Medicine, Osmangazi University, Eskişehir 26040, Turkey; drnkose@gmail.com

**Keywords:** congenital talipes equinovarus, developmental dysplasia of the hip, risk factor, ultrasonographic screening

## Abstract

**Background and Objectives:** Although congenital talipes equinovarus (CTEV) and developmental dysplasia of the hip (DDH) have been associated with certain common intrauterine factors, the relationship between them remains controversial. The aim of this study was to evaluate whether CTEV represents a significant risk factor for DDH. **Materials and Methods:** Data from 3569 newborns who underwent hip ultrasonographic screening between 2010 and 2024 were retrospectively reviewed. Infants diagnosed with true idiopathic CTEV managed with Ponseti serial casting were classified as the “CTEV group”, while secondary cases (e.g., arthrogryposis, neuromuscular conditions) were excluded. DDH was defined as hips initially classified as Graf Type 2a that failed to mature to Type 1 by 3 months, as well as initial Graf Types 2b, 2c, D, 3, or 4. Hips initially classified as Type 2a that spontaneously matured to Type 1 within 3 months without treatment were considered normal. The relationships among sex, presence of CTEV, and DDH were analyzed using Fisher’s exact test and logistic regression analysis. **Results:** Of all participants, 54.7% were female and 45.3% were male. The overall prevalence of DDH was 4.8%. Male sex was identified as a significant protective factor against DDH (*p* < 0.001; OR = 0.32, 95% CI: 0.23–0.47). The presence of CTEV was not identified as a significant risk factor for DDH (OR = 0.86, 95% CI: 0.12–6.45; *p* = 0.884). Stratified analyses by sex also showed no statistically significant association between CTEV and DDH. **Conclusions:** Although CTEV is a distinct foot deformity diagnosed clinically, our findings demonstrate that CTEV was not identified as an independent risk factor for DDH. While these findings do not demonstrate an increased risk of DDH in infants with CTEV, the small number of CTEV cases limits statistical precision, resulting in a wide confidence interval. Therefore, these results should be interpreted with caution, and larger multicenter studies are warranted to clarify whether routine hip ultrasonography can be safely omitted in selective screening pathways.

## 1. Introduction

Congenital talipes equinovarus (CTEV) is one of the most common congenital foot deformities observed in newborns [1]. While prenatal diagnosis via ultrasonography (USG) is occasionally feasible, the deformity is typically clinically evident during routine neonatal physical examinations.

Developmental dysplasia of the hip (DDH) is the most frequent hip pathology encountered during the neonatal period [2]. DDH represents a broad spectrum ranging from mild acetabular dysplasia to subluxation or complete dislocation of the hip joint. While early diagnosis allows successful treatment using non-invasive methods such as the Pavlik harness, delayed or inadequate treatment may necessitate major surgical interventions such as osteotomies [3].

Ultrasonographic evaluation has become the cornerstone of early DDH detection, implemented either through universal screening or selectively in neonates exhibiting established risk factors. While numerous risk factors have been identified, a higher prevalence of DDH has been suggested in infants with CTEV, potentially owing to a shared etiologic mechanism involving intrauterine compression [4]. A recent meta-analysis published in 2025, including 20 studies with 64,583 newborns, identified breech presentation and family history as the most significant risk factors, followed by oligohydramnios, female sex, and high birth weight [5]. However, due to inconsistent evidence in the literature, CTEV was not firmly established as a primary risk factor, indicating that further targeted investigation was warranted.

The association between CTEV and DDH remains controversial and has not been clearly established in the literature. Validating CTEV as an independent risk factor for DDH could impose substantial operational workloads and an economic burden on healthcare systems utilizing selective screening pathways. Conversely, confirming a readily identifiable risk factor could mitigate long-term morbidity in a substantial number of neonates. Therefore, investigating the potential association between CTEV and DDH is of diagnostic and strategic importance for optimizing screening protocols. We hypothesized that true idiopathic CTEV is not an independent risk factor for DDH; clarifying this relationship is essential, as it directly impacts clinical decision-making and screening protocols.

## 2. Materials and Methods

This retrospective study was approved by the Institutional Ethics Committee of Eskişehir Osmangazi University (Decision No: 2025-135, Date: 28 May 2025). We reviewed the medical records of patients who presented between 2010 and 2024 to a tertiary university hospital, which serves as a regional referral center for pediatric orthopedics.

CTEV group: Infants diagnosed with true idiopathic structural CTEV requiring Ponseti serial casting who underwent DDH screening. Mild, postural, or flexible foot deformities were excluded. Furthermore, secondary clubfeet associated with complex conditions such as arthrogryposis, neuromuscular disorders, chromosomal abnormalities, or skeletal dysplasias were strictly excluded from the study.

DDH group: Defined as infants presenting between the 4th and 6th postnatal weeks with Graf ultrasonographic findings other than Type 1. To maintain diagnostic consistency throughout the study, hips classified as Type 2a during the initial examination were re-evaluated. Hips initially classified as Type 2a that spontaneously matured to Type 1 within the first 3 months without treatment were considered normal (representing physiological maturation delay). Conversely, hips initially categorized as Type 2a that failed to mature to Type 1 by 3 months, as well as those initially presenting as Graf Types 2b, 2c, D, 3, or 4, were defined as DDH.

**Figure 1:**
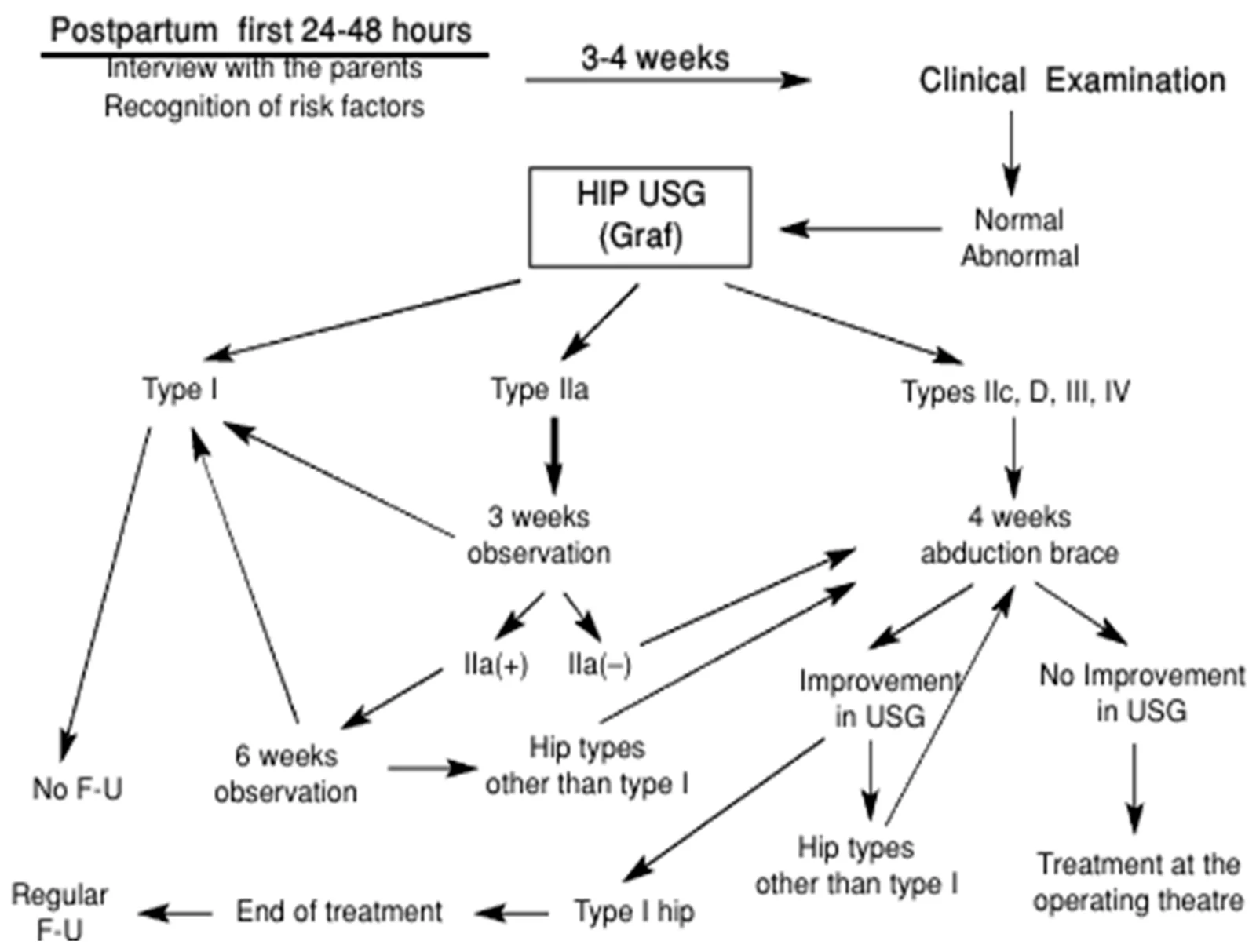
Newborn hip screening and management algorithm [7].

**Figure 2:**
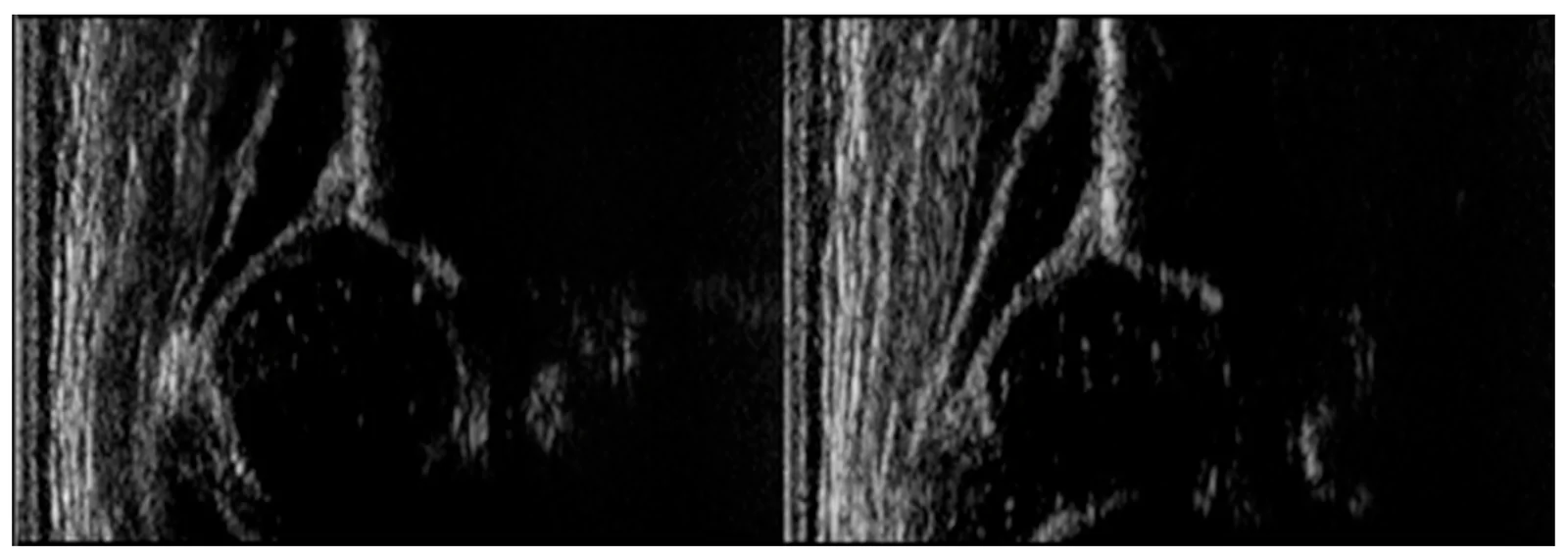
Representative hip ultrasonography examples from the study cohort.

Hip development and maturity were quantified using the Graf ultrasonographic classification system (Samsung HS50, Seoul, Republic of Korea) and its corresponding maturation curve [6], as summarized in Table 1. Ultrasonographic evaluations were conducted by orthopedic clinicians under the direct supervision of a senior pediatric orthopedic specialist. For premature infants (defined as those born before 37 weeks of gestation), the screening schedule was adjusted based on their corrected gestational age, with examinations typically performed between the 4th and 6th postnatal weeks. Neonatal screening and clinical management followed a standardized institutional pathway (Figure 1). Representative coronal ultrasonographs illustrating standard hip USG evaluations performed in our department are presented in Figure 2.

Patients exhibiting an overlap between both groups were identified and thoroughly evaluated for concurrent diagnoses and comorbidities. Conversely, cases with documented neurological or metabolic disorders were excluded from the study.

**Statistical Analysis:** Data analyses were performed using IBM SPSS Statistics version 31 (IBM Corp., Armonk, NY, USA). Descriptive statistics for categorical variables were presented as frequencies (*n*) and percentages (%). The association between the presence of DDH and the frequency of CTEV was evaluated using Fisher’s exact test stratified by sex, and odds ratios (ORs) with 95% confidence intervals (CIs) were calculated.

A multivariable logistic regression model was constructed using sex and CTEV diagnosis as independent variables to evaluate their association with DDH. This focused model structure was specifically selected to evaluate the isolated risk of CTEV independent of other classical risk factors and to determine whether biological sex modulates this relationship (i.e., whether CTEV confers differential risk in male versus female infants). Although data regarding other established risk factors (such as breech presentation and family history) were recorded in our institutional database, only a single infant diagnosed with both CTEV and DDH possessed an additional classical risk factor (breech presentation), indicating that residual confounding from these factors was minimal in our cohort. The goodness of fit of the logistic regression model was assessed using the Hosmer–Lemeshow test.

## 3. Results

A total of 3569 cases were evaluated in this study, of which 54.7% were female and 45.3% were male. The overall prevalence of CTEV was 0.7%, while a diagnosis of DDH was identified in 4.8% of the total cohort (Table 2).

The multivariable logistic regression model (Table 3) demonstrated that male sex was significantly associated with a lower likelihood of DDH compared to female sex (OR = 0.32; 95% CI: 0.23–0.47; *p* < 0.001). However, CTEV was not identified as a statistically significant independent predictor of DDH (OR = 0.86; 95% CI: 0.12–6.45; *p* = 0.884), although the wide confidence interval reflects statistical imprecision due to the low number of CTEV cases.

Sex-stratified analysis (Table 4) revealed no statistically significant association between DDH and CTEV prevalence. Among male infants, CTEV was absent (0%) in the DDH group compared to a 0.9% prevalence in those without DDH, showing no significant difference (*p* > 0.99). Similarly, among female infants, CTEV prevalence was 0.7% in the DDH cohort and 0.6% in the non-DDH cohort, a difference that also lacked statistical significance (OR = 1.24; 95% CI: 0.16–9.65; *p* = 0.575). Overall, no meaningful trend was observed to suggest that CTEV alters the risk of developing DDH.

## 4. Discussion

Regardless of baseline risk factors, we routinely perform hip ultrasonography between the 4th and 6th postnatal weeks on all newborns presenting to our clinic or referred to us for suspected DDH. Through this screening approach, we detected a DDH prevalence of 4.8%. This finding contrasts with a selective screening study of 60,844 children in Norway, which documented a rate of roughly 1 per 1000 live births [8].

Several clinical and methodological factors may account for the observed DDH prevalence of 4.8% in our cohort. As a tertiary university hospital serving as a regional pediatric orthopedic referral center, our institution receives a higher proportion of infants referred for suspected hip abnormalities or clinical risk factors. Furthermore, while clinical guidelines such as those from the American Academy of Orthopedic Surgeons (AAOS) recommend limiting ultrasonography to infants with established risk factors [9], our institution applies universal screening to all presenting newborns. Although hips with transient Graf Type 2a immaturity that spontaneously matured to Type 1 within 3 months were defined as normal and excluded from the DDH cohort, our universal screening strategy enabled the early detection of subclinical pathological dysplasia—such as Graf Type 2b hips—in infants who exhibited normal physical examinations and no traditional risk factors and who would have otherwise been missed under selective screening protocols. Although literature comparing universal and selective screening strategies shows varying detection profiles [10], our observed 4.8% prevalence reflects both the specialized referral pattern of our tertiary center and the identification of true pathological or persistent dysplasia that selective pathways might overlook.

In our study, the prevalence of DDH was 6.8% in females and 2.3% in males. Male sex was identified as a statistically significant protective factor against DDH (*p* < 0.001, OR = 0.32). Large-scale studies from Norway, including more than 100,000 children, and from South Korea, covering 2.5 million children, have similarly reported that female sex is an independent risk factor for DDH [11,12]. Our findings are reaffirming the well-established gender predisposition in DDH.

Among the 3569 cases included in our study, CTEV was detected in 0.7% of newborns. The prevalence of CTEV has been reported in the literature to range between 1% and 3% [13,14], and our results are largely consistent with these published data.

While our findings reaffirm that female sex was significantly associated with an increased risk of DDH, the primary focus was the co-occurrence of CTEV and DDH. When the prevalence of CTEV was evaluated separately based on DDH status, the rate of CTEV among males was 0.9% in those without DDH and 0% in those diagnosed with DDH. Although this distribution suggests a potential inverse relationship between CTEV and DDH in male infants, the limited number of cases precluded a definitive statistical comparison.

Among female infants, the prevalence of CTEV was similar between the DDH and non-DDH groups, indicating no association between CTEV and altered risk of DDH.

Taken together, these findings imply that although female sex remains a well-recognized risk factor for DDH, the concomitant presence of CTEV does not appear to contribute to any additive risk for DDH.

Unnecessary USG may increase healthcare workload without improving clinical outcomes. Therefore, incorporating CTEV as a stand-alone indication for routine ultrasound evaluation may not yield any substantial clinical benefit.

The relationship between CTEV and DDH remains controversial in the literature. A meta-analysis by De Hundt et al. found no significant association between foot deformities and DDH [15]. Similarly, a systematic review and meta-analysis conducted by Ibrahim et al. reported that the prevalence of DDH among individuals with CTEV was comparable to the general population; thus, they emphasized that routine hip ultrasonography is not warranted solely based on a diagnosis of CTEV [16]. Conversely, other studies have reported an increased risk of DDH in children with CTEV [17,18,19], with a relative risk reaching as high as 10.34, which is substantially greater than that of the general population. These discrepancies among existing studies are likely influenced by variations in screening protocols, population characteristics, and diagnostic thresholds.

In our study, the prevalence of CTEV was 0.7% in infants without DDH and 0.6% in those with DDH. Although CTEV appeared to have a slightly protective trend, this difference was not statistically significant (*p* = 1.00, OR = 0.79).

Overall, while our findings did not detect a statistically significant association between CTEV and DDH, the statistical imprecision due to the small CTEV sample size precludes a definitive conclusion. Therefore, routine clinical hip examinations remain essential, and larger multicenter cohorts are needed.

**Limitations:** This study has several limitations. First, the number of CTEV cases was relatively small (*n* = 26), which resulted in a wide 95% confidence interval (0.12–6.45) in the multivariable analysis. Consequently, while no statistically significant association was detected, the precision of the estimate is limited, and a minor clinical risk cannot be definitively ruled out. Second, the single-center design of our study may limit the generalizability of our findings to broader populations with different geographic or healthcare profiles.

## 5. Conclusions

In conclusion, our study did not demonstrate a statistically significant association between true idiopathic CTEV and DDH. However, given the relatively small sample size of CTEV cases and the resulting statistical uncertainty, these findings should be interpreted with caution. While these results suggest that CTEV may not represent an isolated risk factor for DDH, routine clinical hip examinations remain essential, and larger multicenter prospective cohorts are required to confirm whether routine ultrasound screening can be safely omitted in selective screening protocols.

## Figures and Tables

**Table 1:** Ultrasonographic hip typing according to the Graf’s classification system.

Type	Description	α Angle (°)	β Angle (°)
**I**	Mature (normal) hip	≥60	Ia: <55
			Ib: ≥55
**IIa**	Physiological delay in maturation (≤3 months of age)	50–59	55-77
**IIb**	Pathological delay in maturation (>3 months of age)	50–59	55-77
**IIc**	At-risk or critical hip	43–49	≤77
**D**	Hip on the point of dislocation (decentric)	<43	>77
**III**	Dislocated hip	<43	>77
	III a: No disturbance in the structure of the cartilaginous acetabular roof		
	III b: Disturbance in the structure of the cartilaginous acetabular roof		
**IV**	Highly dislocated hip	<43	>77

**Table 2:** Descriptive characteristics of the study population.

Variables		*n* (%)
Sex	Female	1954 (54.7)
	Male	1615 (45.3)
Presence of CTEV	Present	26 (0.7)
	Absent	3543 (99.3)
Diagnosis of DDH	Present	172 (4.8)
	Absent	3397 (95.2)

CTEV: Congenital talipes equinovarus; DDH: Developmental dysplasia of the hip.

**Table 3:** Logistic regression analysis for factors associated with developmental dysplasia of the hip (DDH).

Independent Variables	OR (95% CI)	*p*-Value
Sex (Male vs. Female)	0.32 (0.23–0.47)	<0.001
CTEV diagnosis	0.86 (0.12–6.45)	0.884

CTEV: Congenital talipes equinovarus; OR: Odds ratio; CI: Confidence interval.

**Table 4:** Comparison of CTEV prevalence according to the presence of DDH by sex.

Sex	DDH Status	CTEV Absent *n* (%)	*p*-Value *	OR (95%Cl)
**Males**	No DDH	1563 (99.1)	>0.999	N/A
	With DDH	38 (100.0)		
**Females**	No DDH	1809 (99.4)	0.575	1.24 (0.16–9.65)
	With DDH	133 (99.3)		

* Fischer’s exact test. CTEV: Congenital talipes equinovarus; DDH: Developmental dysplasia of the hip; OR: Odds ratio; CI: Confidence interval.

## Data Availability

The data presented in this study are available on request from the corresponding author. The data are not publicly available due to privacy and ethical restrictions regarding retrospective patient medical records.

## Abbreviations

The following abbreviations are used in this manuscript:

CTEV	Congenital Talipes Equinovarus
DDH	Developmental Dysplasia of the Hip
USG	Ultrasonography
OR	Odds Ratio
